# Real-time Point-of-care Ultrasound for the Diagnosis and Treatment of Testicular Torsion

**DOI:** 10.24908/pocus.v6i2.15186

**Published:** 2021-11-23

**Authors:** Rahul V Nene, Rachna Subramony, Michael Macias, Colleen Campbell, Amir Aminlari

**Affiliations:** 1 Department of Emergency Medicine, University of California San Diego, CA

**Keywords:** point-of-care ultrasound, emergency medicine, acute scrotal pain, testicular torsion

## Abstract

**Background**: Testicular torsion is a surgical emergency that needs prompt diagnosis and treatment. Point-of-Care ultrasound (POCUS) can not only establish the diagnosis but also guide the Emergency Physician in evaluating the response to manual detorsion. **Case Report**: We describe the case of a 13-year-old male who presented with acute scrotal pain. We demonstrate how bedside ultrasound was used to make the diagnosis of testicular torsion, guide the technique for manual detorsion, and confirm adequate return of blood flow. Our case illustrates the ease with which POCUS can be used in real time to diagnose and treat organ-threatening pathology, but more importantly, it shows how real-time POCUS was used to detorse a testicle that was refractory to the standard detorsion technique. **Conclusion**: The acute scrotum is a time-sensitive presentation and if testicular torsion is present, the diagnosis should be made as soon as possible. Many Emergency Departments do not have 24-hour coverage of ultrasound technicians, which would delay the diagnosis and treatment. Moreover, when manual detorsion is attempted, it often does not work because the testicle may need more than the standard 180 degree medial to lateral rotation. POCUS provides real-time analysis of return of blood flow and can thus guide further rotation, or opposite direction rotation, as needed.

## Introduction

Testicular torsion is a surgical emergency with a yearly incidence of 3.8 per 100,000 males under the age of 18 1. The morbidity associated with testicular torsion is significant as 42% of surgeries result in orchiectomy 1. However, testicular salvage rates are 90% to 100% if intervention is performed within 6 hours of symptom onset 2. Thus, prompt diagnosis and treatment are critical in preventing testicular ischemic damage or necrosis. 

Pre-operative manual detorsion is the fastest way to restore blood flow to the scrotum. This maneuver involves the physical rotation of the affected testicle in the opposite direction of the torsion, most commonly medial to lateral (“open book”). Manual detorsion can improve testicular salvage, however there is wide variation in the reported success of this strategy (26-95% successful) 3, 4, 5, 6.Success of manual detorsion can be complicated by unclear direction and degree of cord rotation 4. Point-of-care ultrasound (POCUS) can be used by emergency physicians to detect testicular torsion. POCUS has a high sensitivity and specificity in the diagnosis of testicular torsion and its implementation in the work-up for acute scrotal pain has been reported to decrease the time to intervention 7, 8, 9. High accuracy in determining direction of cord twist by ultrasound has also been reported 10. Here we report the use of point-of-care ultrasound (POCUS) to guide manual detorsion. 

## Case Presentation

**Figure 1:**
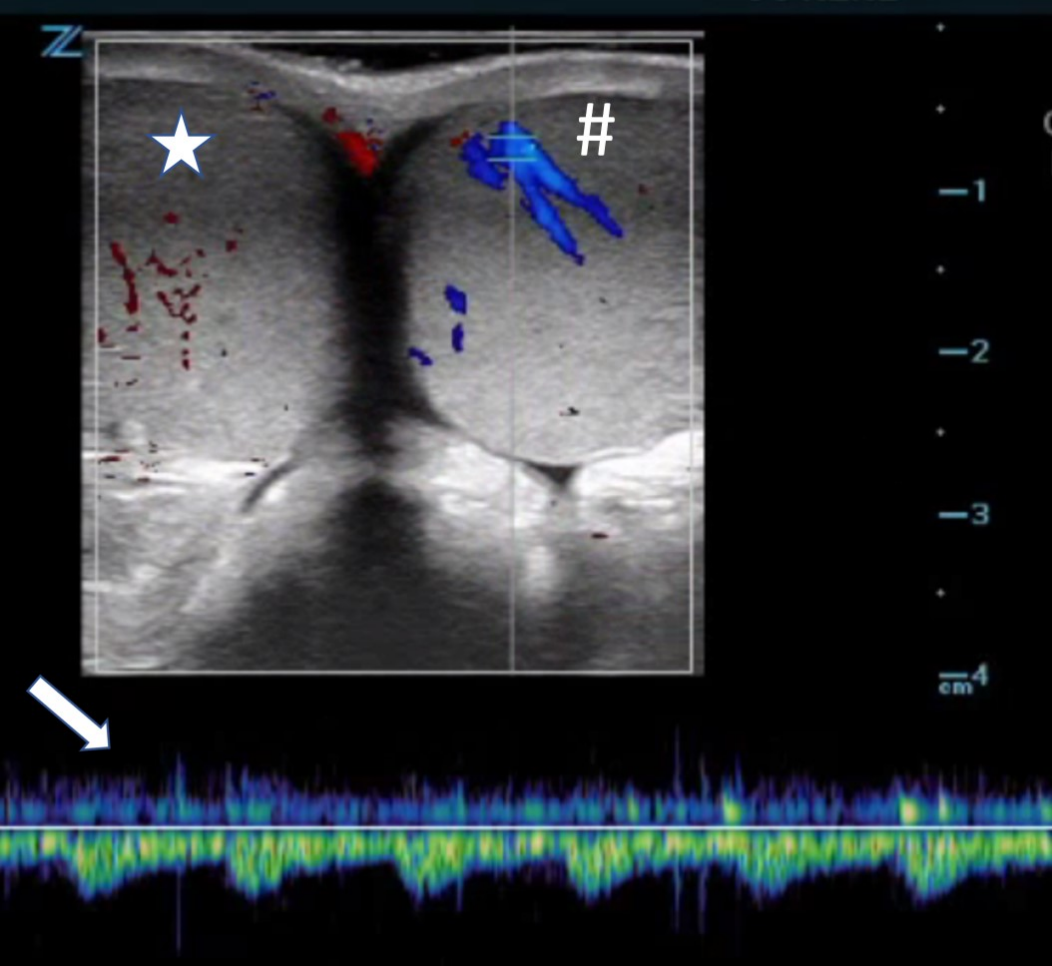
Point-of-care testicular ultrasound using doppler ultrasound imaging - Appropriate flow is demonstrated to the left testicle (#) indicated by color signal and arterial waveform on pulse wave doppler (arrow). No consistent color signal is appreciated over the right testicle (star), confirming the diagnosis of testicular torsion.

A 13-year-old healthy male presented to the emergency department with acute onset of atraumatic right testicular pain 2 hours prior to arrival. He was afebrile and hemodynamically stable. Genital exam was remarkable for a firm high-riding right testicle, which was diffusely tender to palpation without overlying skin changes. Point-of-care ultrasound (POCUS) was performed using a high-frequency linear probe, which was placed in the transverse axis along the scrotum to obtain an image where both testicles are visible side by side (Figure 1). Color and pulse-wave doppler were used to evaluate testicular blood flow, demonstrating lack of flow to the right testicle, confirming the diagnosis of acute testicular torsion.

**Figure 2:**
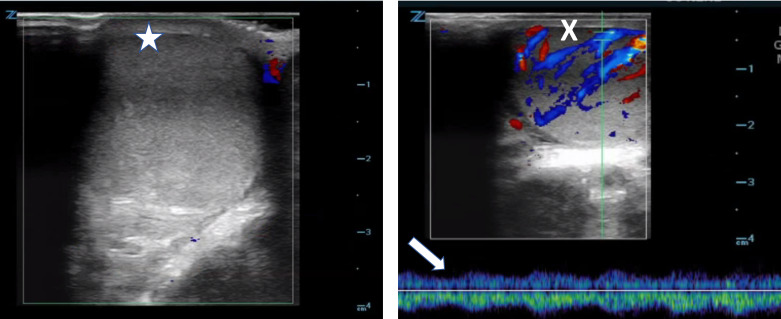
Doppler ultrasound imaging of right testicle during attempted detorsion. A) There was still absence of blood flow after initial detorsion attempt demonstrated by lack of color signal over the right testicle (star). B) After additional detorsion, color signal is now visible over the right testicle (X) and return of arterial waveform on pulse wave doppler (arrow), indicating restoration of blood flow.

Manual detorsion was then attempted, initially by twisting the right testicle medial-to-lateral on the vascular pedicle 180 degrees. Bedside ultrasound was used to reevaluate the testicle and it continued to demonstrate a lack of flow (Figure 2A). The testicle was then rotated an additional 90 degrees, after which the patient had immediate relief of his pain and the testicle no longer felt as firm. Bedside ultrasound confirmed return of blood flow to the testicle (Figure 2B). The urology service was consulted, and the patient was taken to the operating room for emergent orchiopexy.

## Discussion

Testicular torsion is a surgical emergency and prompt diagnosis and treatment is necessary for testicular salvage 11.Classic exam findings include high-riding testis with profound swelling, tenderness, and loss of the cremasteric reflex. POCUS can help to rapidly make this diagnosis. Characteristic findings include loss of visual color flow to the affected testicle, spectral Doppler showing a high resistance arterial pattern, heterogenous echotexture and enlargement of the affected testis, and the “whirlpool sign” with visible twisting of the spermatic cord 12.Manual detorsion can be attempted by “opening the book” with medial-to-lateral rotation. However, this is not always effective, as further rotation, or even rotation in the opposite direction may be required to effectively de-torse the testis. Real time ultrasound can be used to assess for improvement in flow during the procedure, specifically by guiding rotation degree and direction. 

The diagnostic accuracy of POCUS for testicular torsion when performed by emergency physicians is 95% sensitive and 94% specific 8. It has also been shown to be accurate for detecting torsion in children when performed by pediatric emergency physicians 7. Despite the fact that POCUS is listed in the “Model of the Clinical Practice of Emergency Medicine as an integral diagnostic procedure,” the exact method and applications differ from program to program and the required “didactic, hands-on, and experiential components” of emergency ultrasound are not specifically outlined by the Residency Review Committee for Emergency Medicine (RRC-EM) or any single sponsoring group 13. This leads to a variation in experience and knowledge with image acquisition and interpretation by emergency physicians, however studies have shown that it can be taught to emergency physicians using a condensed program 14. Despite adequate training, it is important to be aware of the pitfalls when performing POC testicular ultrasound that could lead to a misdiagnosis. For example, the use of color doppler ultrasound can have false negatives, especially in the case of partial torsion when there is arterial flow present but no venous flow, so it is crucial to check for both arterial and venous flow when performing this study. In some cases, arterial flow may be present but may show a high-resistance pattern, which can be nondiagnostic. Therefore, it is important to always consult the urology service when the clinical presentation is concerning for torsion even with a negative ultrasound.

## Statement of ethics approval/consent

We attest that our institution does not require IRB approval for case reports or de-identified clinical images and that the appropriate consent for the use of these images has been obtained from the patient/legal guardian. All authors reviewed and assisted with revisions of the final manuscript. RVN takes responsibility for the manuscript as a whole. 

## Financial Support

None
